# Laryngectomy‐Sparing Radio Frequency Ablation for Laryngeal Metastasis in Recurrent Papillary Thyroid Carcinoma: A Case Report

**DOI:** 10.1155/crie/6647587

**Published:** 2026-02-22

**Authors:** Hossein Chegeni, Hojat Ebrahiminik, Haleh Chehrehgosha, Maryam Pourashraf, Hossein Parsa, Zeinab Azizi, Seyed Hossein Samadanifard, Vahan Moradians, Ebrahim Karimi

**Affiliations:** ^1^ Department of Radiology, Tehran University of Medical Sciences, Tehran, Tehran Province, Iran, tums.ac.ir; ^2^ Department of Interventional Radiology and Radiation Sciences Research Center, AJA University of Medical Sciences, Tehran, Tehran Province, Iran, ajaums.ac.ir; ^3^ Department of Endocrinology, Hazrat Rasool Hospital, School of Medicine, Iran University of Medical Sciences, Tehran, Iran, iums.ac.ir; ^4^ Professor of General and Vascular Surgery, Qazvin University of Medical Science, Qazvin, Iran, qums.ac.ir; ^5^ Hazrat Rasool Hospital, School of Medicine, Iran University of Medical Sciences, Tehran, Iran, iums.ac.ir; ^6^ Otorhinolaryngology Research Center, Tehran University of Medical Sciences, Tehran, Iran, tums.ac.ir

**Keywords:** case report, laryngeal metastasis, radio frequency ablation, recurrent papillary thyroid carcinoma

## Abstract

**Introduction:**

Recurrence rate of papillary thyroid carcinoma (PTC) due to laryngeal metastasis is rare, radio frequency ablation (RFA) shows efficacy in management of recurrent PTC. This case is important due to its rarity and limited evidence on using RFA for recurrent PTC with laryngeal metastasis, focusing on larynx preservation and voice maintenance.

**Case Report:**

A 64‐year‐old woman with recurrent PTC experienced shortness of breath, hoarseness, and painful swallowing. Imaging revealed two masses affecting her vocal cords and severe airway narrowing. After declining a laryngectomy, she underwent RFA and temporary tracheostomy. Follow‐up showed successful treatment, with no residual mass, and her voice improved.

**Conclusion:**

This case shows a successful performance of RFA in a patient with recurrent and invasive PTC with laryngeal involvement who refused laryngectomy. It seems that combining RFA with radioactive iodine and external beam radiation can be considered in patients with recurrent PTC with laryngeal metastasis to maintain voice.

## 1. Introduction

The recurrence rates of papillary thyroid carcinoma (PTC) range from 6.6% to 28%, depending on factors like initial cancer stage, tumor size, lymph node involvement, patient age, and surgery thoroughness [1, 2].

Laryngeal metastasis in PTC is rare, occurring in less than 1% of cases, with cancer mainly spreading to local lymph nodes or distant sites like bones and lungs. When it does happen, it often indicates advanced disease and may lead to difficulty in breathing, speech, and swallowing. Timely diagnosis and intervention are crucial, with surgical options including peeling, partial laryngectomy, or total laryngectomy [3–6].

Studies have substantiated the clinical efficacy of radio frequency ablation (RFA) in management of recurrent PTC. The procedure has shown favorable outcomes in terms of local tumor control and improvement of prognosis. RFA presents several potential advantages, including reduced invasiveness, diminished morbidity, brief procedural times, and low complication rates [7–9].

This case report describes the application of RFA for recurrent PTC involving the larynx, which has yielded favorable outcomes during follow‐up evaluations.

## 2. Case Presentation

A 64‐year‐old woman with a history of recurrent PTC was referred to the clinic with complaint of shortness of breath, hoarseness, and painful swallowing. She had undergone four surgical interventions and had received a total cumulative dosage of 600 mCi of radioactive iodine therapy.

Serum thyroglobulin (Tg) concentration was 50 ng/mL. As shown in Figure 1, neck computed tomography (CT) scan and ultrasound (US) showed two masses: one was 30 mm × 23 mm × 27 mm (10 mL) in the right vocal cord, affecting the thyroid cartilage and strap muscles, causing severe airway narrowing. The second mass was 30 mm × 18 mm × 24 mm (6.7 mL) in the left strap muscle. A 12 mm × 11 mm × 11 mm (1 mL) metastatic lymph node was found in the right paratracheal space (Figure 1). Fine needle aspiration and Tg washout concentration confirmed PTC metastasis. The laryngoscopy showed significant airway narrowing and total paralysis of the right vocal cord.

Figure 1Preablation findings of this patient. (A) There is a visible anterior neck mass with skin involvement. (B, C) Preablation neck CT scan showing an enhancing laryngeal mass causing severe airway narrowing. (D, E) Preablation gray‐scale and color doppler thyroid and neck US showing lobulated hypoechoic mass with size of 30 mm × 23 mm × 27 mm (10 mL) in anterior of trachea, at site of previous thyroidectomy with peripheral and central vascularity.

**Figure:**
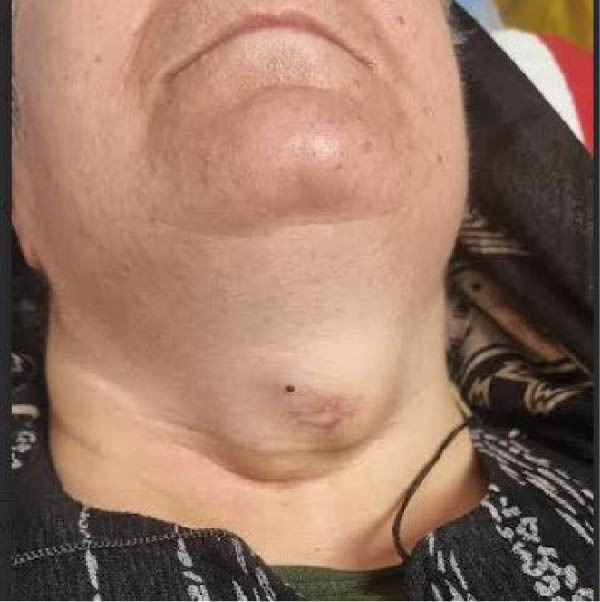
(A)

**Figure:**
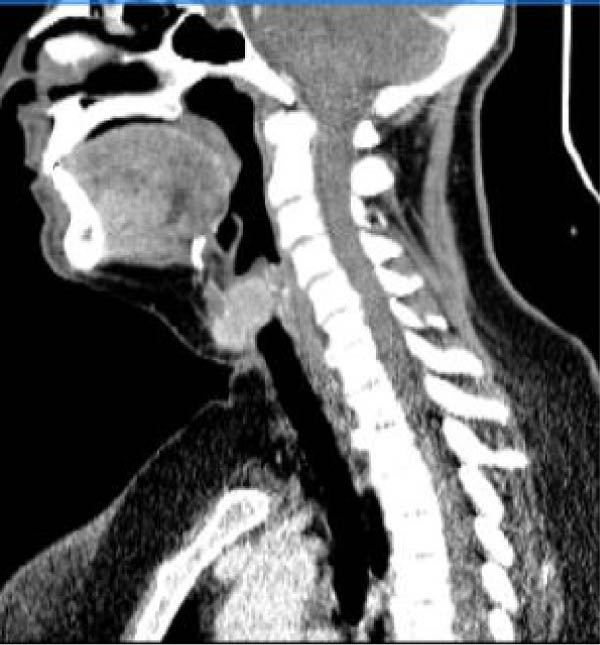
(B)

**Figure:**
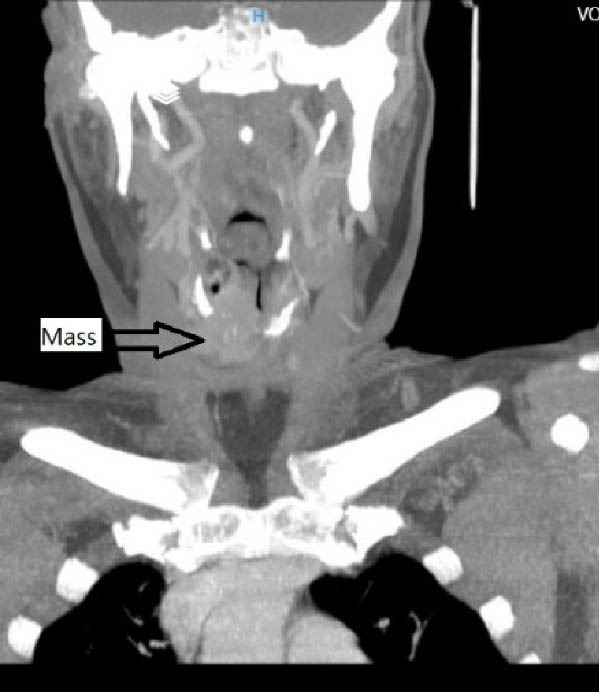
(C)

**Figure:**
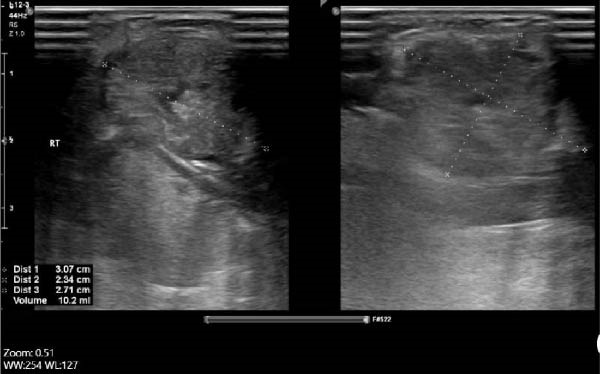
(D)

**Figure:**
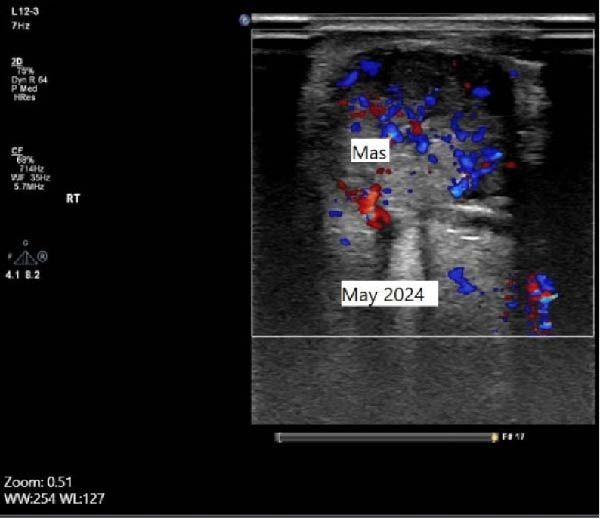
(E)

During this period, the patient developed low‐grade B‐cell lymphoma, possibly due to high radioiodine exposure. Additional radioiodine ablation was not advisable. A laryngectomy was recommended, but she declined due to fear of losing her voice. The physician referred her to our clinic for consultation on RFA feasibility.

**Figure 2:**
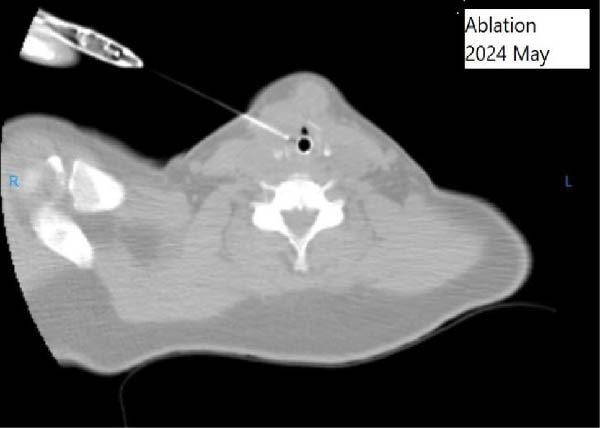
During RFA, CT‐guided ablation of the laryngeal mass.

The patient was admitted to the hospital, and the RFA was performed utilizing CT scan guidance, subsequently followed by a tracheostomy for safety. Lesions were identified using US and CT fluoroscopy. Following prepping and draping the site, local and general anesthesia, and hydro‐dissection with dextrose water, these lesions were ablated utilizing 10‐mm active‐tips radio frequency needle and 50‐W power using moving shot technique; additionally, unlike standard thyroid RFA protocols, we administrated one cc of 99% ethanol alongside RFA (Figure 2). Adjunctive ethanol injection was performed to enhance efficacy and reduce recurrence complication rate, particularly given the lesion’s proximity to the laryngeal mucosa where complete thermal ablation might be challenging. A temporary tracheostomy was performed to manage pronounced airway stenosis, anticipated postoperative edema, and to mitigate the risk of aspiration pneumonia. No complication ensued during the ablation procedure; however, the patient was admitted to intensive care unit due to aspiration pneumonia. The patient was discharged 7 days after the procedure.

**Figure 4:**
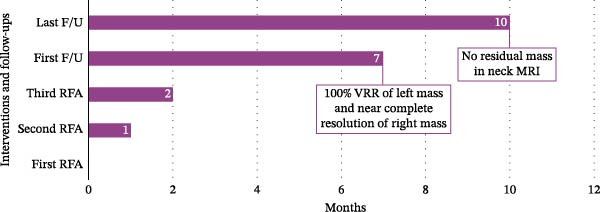
The timeline of interventions and follow‐ups. F/U, follow‐up; MRI, magnetic resonance imaging; RFA, radio frequency ablation; VRR, volume reduction rate.

The Color Doppler US exam showed some areas not treated, so RFA was done again in two other sessions, spaced about 1 month apart, for complete treatment. In comparison with the initial session, which utilized a 10‐mm active‐tip electrode and 50‐W power to cover the larger laryngeal mass, the subsequent sessions employed smaller 5‐mm electrodes and lower power (40‐W) to allow more precise ablation near critical structures. The patient underwent three ablation sessions in total. Two months after the first ablation, laryngoscopy confirmed airway patency, and the tracheostomy was closed. At the 7‐month follow‐up, the serum Tg level was steady at 42.36 ng/mL. A CT scan with intravenous contrast revealed a 100% volume reduction rate (VRR) of left mass and metastatic lymph node compared to pre‐ablation imaging. The right laryngeal mass was not detected, although a small part of this area showed mucosal irregularity and post contrast enhancement and it means that near complete resolution was achieved in images. No lymphadenopathy was observed. At the last follow‐up, 10 months after the initial ablation, neck magnetic resonance imaging (MRI) indicated no residual mass (Figure 3). Laryngoscopy showed paralysis of the right vocal cord, with the left cord compensating. The patient can speak well and has no issues with swallowing. She is now under careful observation. The timeline of interventions and follow‐up is summarized in Figure 4.

Figure 3Postablation findings of the laryngeal masses in the patient with laryngeal mass due to PTC recurrence 10 months after the initial ablation. Ten months postablation contrast‐enhanced CT scan (A, B) and contrast‐enhanced MRI (C, D) revealed no laryngeal mass or lymphadenopathy.

**Figure:**
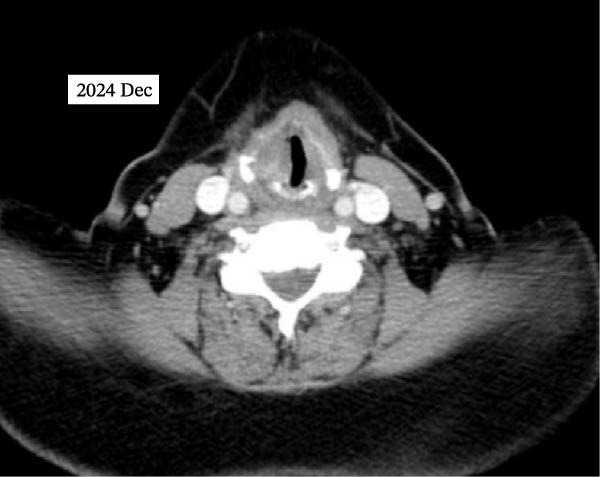
(A)

**Figure:**
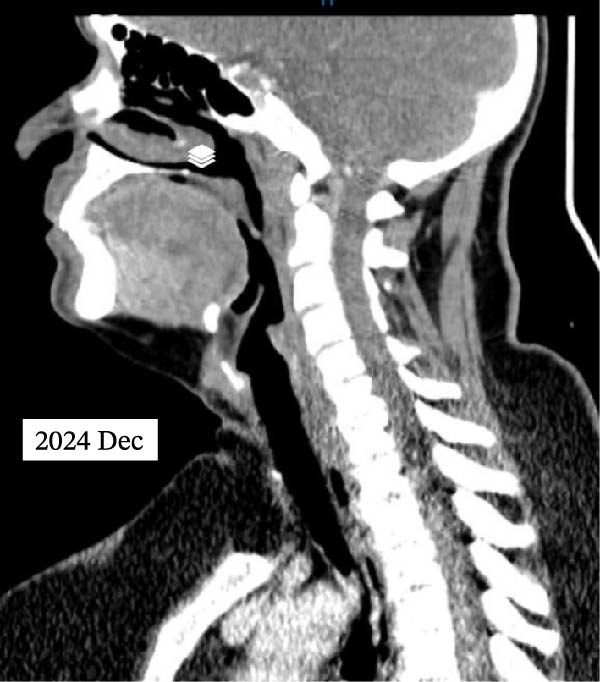
(B)

**Figure:**
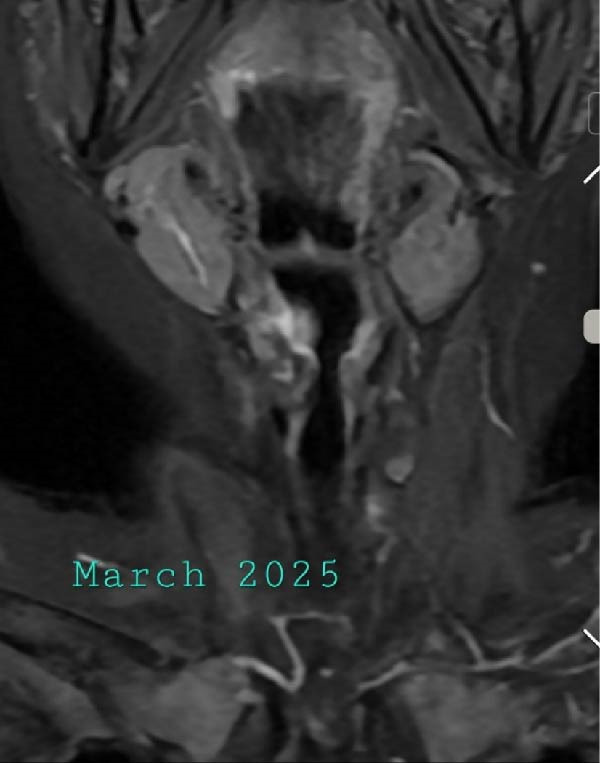
(C)

**Figure:**
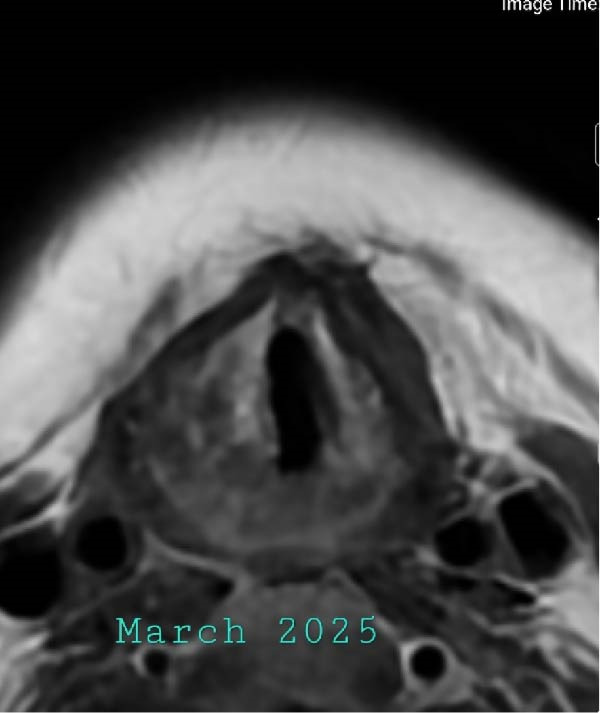
(D)

## 3. Discussion

Laryngeal metastasis from PTC is extremely rare. There are two main hypotheses regarding the mechanisms of laryngeal involvement. The first mechanism is direct invasion to larynx, especially in cases with extrathyroidal extension. The second one is hematogenous or lymphatic spread to the larynx. Laryngeal involvement in PTC can lead to significant symptoms, including difficulty swallowing and breathing [5, 6]. Management strategies for patients with recurrent PTC include surgery, radioiodine ablation, radiotherapy, and the use of tyrosine kinase inhibitors [7–9].

Our case received a high cumulative dose of radioactive iodine and opted against laryngectomy, despite being a suitable candidate for total laryngectomy due to the extensive laryngeal involvement. Additionally, the use of tyrosine kinase inhibitors was not feasible in this case because of a lymphoproliferative disorder [10]. She underwent three sessions of RFA, which successfully preserved her larynx, led to substantial tumor reduction, alleviated symptoms, and improved her quality of life.

Other studies have explored the effectiveness of RFA in patients with recurrent PTC. A meta‐analysis showed a tumor VRR between 89.5% and 100%, a complete disappearance rate of 68.8%, and an average of 1.3 ablation sessions required [11]. This indicates that RFA is a viable option for patients who are either ineligible for surgery or choose to decline it [8, 11]. Another study demonstrated that RFA can yield both structural and biochemical improvements in locoregionally recurrent thyroid cancer, with a low complication rate [9]. In 2024, findings highlighted high recurrence‐free survival rates with RFA for recurrent differentiated thyroid cancer, supporting its use for local control of recurrent PTCs. However, to our knowledge, there are no documented cases of recurrent PTC with laryngeal metastasis treated with RFA [10].

Recent clinical guidelines and expert consensus highlight the growing role of RFA as a safe and effective minimally invasive option for managing head and neck malignancies, especially in patients with locoregional recurrence who are not eligible for surgery or systemic therapy [12, 13].

It is important to establish clear selection criteria for RFA to guide its use in comparable clinical scenarios. Ideal candidates may include those with localized recurrence, contraindications to surgery or tyrosine kinase inhibitors, and preserved organ function. Additionally, long‐term monitoring should involve serial imaging, Tg levels, and symptom assessment to detect recurrence early and guide further intervention. We believe that the multidisciplinary tumor board can facilitate collaborative decision‐making, allowing various specialists to discuss risks and benefits of nonsurgical management and achieve consensus on the most appropriate treatment plan for the patient. It seems image‐guided ablation offers a promising organ‐sparing option in oncology, enabling precise tumor targeting while preserving function. Its role is expected to expand with advances in imaging and minimally invasive techniques.

## 4. Conclusion

This case is important because it is rare and has little evidence about using RFA for recurrent PTC with laryngeal metastasis, especially for preserving the larynx and maintaining voice. Using RFA with other treatments like radioactive iodine therapy and external beam radiation therapy can be considered in patients with recurrent PTC with laryngeal metastasis.

## Author Contributions

Conceptualization: Hossein Chegeni. Data curation, investigation, writing – review and editing: Hossein Chegeni, Hojat Ebrahiminik, Haleh Chehrehgosha, Maryam Pourashraf, Hossein Parsa, Zeinab Azizi, Seyed Hossein Samadanifard, Vahan Moradians, and Ebrahim Karimi. Resources, supervision, validation: Hojat Ebrahiminik and Hossein Chegeni. V: Hojat Ebrahiminik, Hossein Chegeni. Writing – original draft: Hossein Chegeni and Haleh Chehrehgosha.

## Funding

No funding was received for this manuscript.

## Ethics Statement

All procedures performed in studies involving human participants were in accordance with the ethical standards of the institutional and/or national research committee and with the 1964 Helsinki Declaration and its later amendments or comparable ethical standards. Also, the study was approved by Ethics Committee of the TIRAD Imaging Institute.

## Consent

The patient is sufficiently anonymized according to the ICMJE guidelines. Written consent was obtained from the patient before ablation.

## Conflicts of Interest

The authors declare no conflicts of interest.

## Data Availability

The data that support the findings of this study are available from the corresponding author upon reasonable request.
